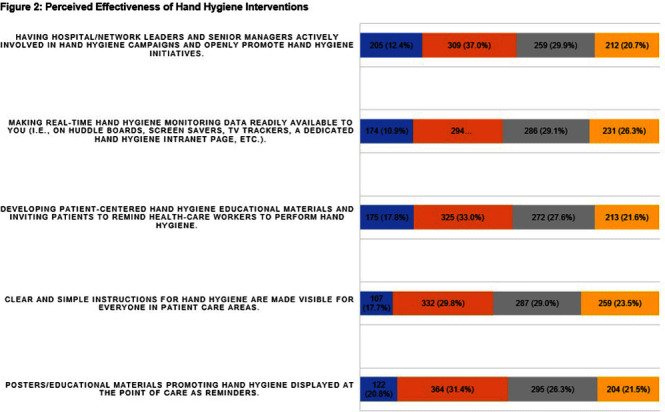# Staff Perspectives on Barriers & Facilitators to Meeting Hand Hygiene Goals in a Multicenter Academic Hospital System

**DOI:** 10.1017/ash.2024.245

**Published:** 2024-09-16

**Authors:** Kevin Gibas, Nathan Kinsella, Parente Stephanie, Megan Diamond, Kerry Blanchard, Leonard Mermel

**Affiliations:** Rhode Island Hospital/Lifespan Hospital System; Lifespan Health System; Warren Alpert Medical School of Brown University

## Abstract

**Background:** Proper hand hygiene is the most important practice to reduce the transmission of infections in healthcare settings. Despite this, healthcare institutions continue to struggle to achieve and maintain high rates of hand hygiene compliance among healthcare workers with some studies estimating national healthcare worker hand hygiene compliance to be approximately 50%. **Methods:** We conducted an anonymous one-time survey of our Lifespan Hospital System employees to evaluate barriers and facilitators to performing hand hygiene as well as interventions to improve hand hygiene compliance. The survey was designed with guidance from the Consolidated Framework for Implementation Research and input from Lifespan infection prevention staff. **Result:** Over four weeks 985 (6%) Lifespan employees completed the survey. Figure 1 shows the aggregate results of the first 4 survey questions which focused on hand hygiene infrastructure at Lifespan, including availability of sanitizer, staff to manage hand hygiene supplies, and educational materials/reminders. One significant finding was >70% of respondents reported that they either did not know if their unit/department has a person assigned to replace/monitor hand hygiene supplies, or if so, who that person is. We also asked employees to rate how effective different interventions would be at improving hand hygiene compliance. Figure 2 shows of five proposed interventions, three were rated as either “moderately effective” or “very effective” by >50% of respondents. These included displaying hand hygiene instructions, making hand hygiene data available to employees, and displaying materials/reminders promoting hand hygiene. There were also 977 free-text responses regarding “barriers or facilitators to proper hand hygiene”. Major barriers identified were a lack of staff to monitor and refill supplies, slow replacement of hand hygiene products, lack of sanitizer dispensers and sinks, inconsistency of sink location and dispenser placement, lack of hand hygiene reminders/educational materials, time constraints, skin irritation from sanitizer, and an inability to have dispensers in behavioral health units. Survey responses led us to enhance the following: educational materials and reminders in work areas; staff education; leadership involvement in hand hygiene initiatives; routine auditing and feedback; conveniently placed sanitizer dispensers and sinks at the point of care; and making hand hygiene compliance data readily available to staff. **Conclusion:** This survey identifies important barriers and facilitators to achieving high rates of hand hygiene compliance among healthcare workers and provides the basis for interventions aimed at improving hand hygiene compliance in a large multicenter academic hospital system.

**Figure:**
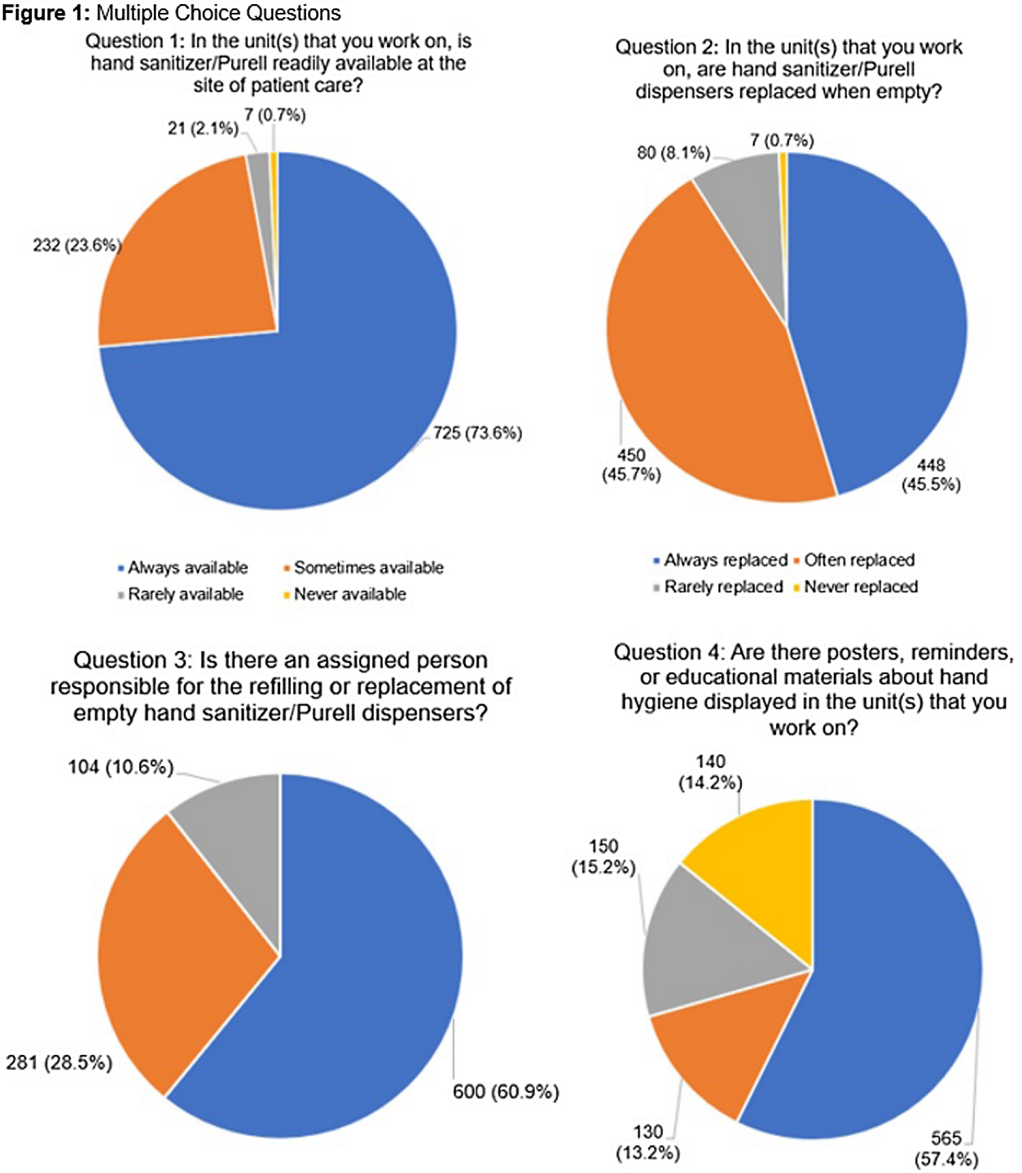

**Figure:**